# p53 controls colorectal cancer cell invasion by inhibiting the NF-κB-mediated activation of Fascin

**DOI:** 10.18632/oncotarget.5137

**Published:** 2015-08-29

**Authors:** Xinbing Sui, Jing Zhu, Haimei Tang, Chan Wang, Jichun Zhou, Weidong Han, Xian Wang, Yong Fang, Yinghua Xu, Da Li, Rui Chen, Junhong Ma, Zhao Jing, Xidong Gu, Hongming Pan, Chao He

**Affiliations:** ^1^ Department of Medical Oncology, Sir Run Run Shaw Hospital, Zhejiang University, Hangzhou, Zhejiang, China; ^2^ Biomedical Research Center and Key Laboratory of Biotherapy of Zhejiang Province, Zhejiang University, Hangzhou, Zhejiang, China; ^3^ Department of Colorectal Surgery, Sir Run Run Shaw Hospital, Zhejiang University, Hangzhou, Zhejiang, China; ^4^ Department of Surgical Oncology, Sir Run Run Shaw Hospital, Zhejiang University, Hangzhou, Zhejiang, China; ^5^ Department of Breast Surgery, the First Affiliated Hospital of Zhejiang Chinese Medical University, Hangzhou, China; ^6^ Department of Gastrointestinal Surgery, Nankai Hospital, Nankai District, Tianjin, China

**Keywords:** p53, cancer, cell invasion, NF-κBF, Fascin

## Abstract

p53 mutation is known to contribute to cancer progression. Fascin is an actin-bundling protein and has been recently identified to promote cancer cell migration and invasion through its role in formation of cellular protrusions such as filopodia and invadopodia. However, the relationship between p53 and Fascin is not understood. Here, we have found a new link between them. In colorectal adenocarcinomas, p53 mutation correlated with high NF-κB, Fascin and low E-cadherin expression. Moreover, this expression profile was shown to contribute to poor overall survival in patients with colorectal cancer. Wild-type p53 could inhibit NF-κB activity that repressed the expression of Fascin and cancer cell invasiveness. In contrast, in p53-deficient primary cultured cells, NF-κB activity was enhanced and then activation of NF-κB increased the expression of Fascin. In further analysis, we showed that NF-κB was a key determinant for p53 deletion-stimulated Fascin expression. Inhibition of NF-κB /p65 expression by pharmacological compound or p65 siRNA suppressed Fascin activity in p53-deficient cells. Moreover, restoration of p53 expression decreased the activation of Fascin through suppression of the NF-κB pathway. Taken together, these data suggest that a negative-feedback loop exists, whereby p53 can suppress colorectal cancer cell invasion by inhibiting the NF-κB-mediated activation of Fascin.

## INTRODUCTION

Tumor metastasis, the most common cause of death for the cancer patients, is a multi-step process by which cancer cells disseminate from their primary site and subsequently form secondary distant tumor sites, where they arrest in the bloodstream and reinitiate tumor growth [1, 2]. Although plenty of progresses have been made for the cancer treatment in the past few years, tumor metastasis is still a leading cause of cancer-related mortality.

Epithelial-mesenchymal transition (EMT) is a process, which stimulates epithelial cells to acquire the highly invasive and metastatic properties of mesenchymal cells [3], thus has been demonstrated to play a critical role in promoting cancer metastasis, especially for epithelium-derived carcinoma [4]. A large number of transcription factors are involved in this process. The characteristics of EMT are loss of the epithelial junction molecule E-cadherin and gain of mesenchymal markers such as Snail, Slug, Vimentin, Twist, Zeb, and Sip1 [5, 6]. Among these transcription factors, the high expression of E-cadherin suppresses tumor invasion and metastasis, whereas the downregulation of E-cadherin facilitates malignant transformation and metastatic progression [7, 8].

Fascin is an actin-binding protein that expressed in a large number of human carcinomas and is usually upregulated as an important component of cancer cell epithelial to mesenchymal progression [9, 10]. In normal epithelia, Fascin is usually present at low level or absent, however, its expression is often increased in epithelial neoplasms such as esophageal carcinomas, colorectal adenocarcinomas and other types of cancer [11–13]. Fascin can stabilize actin bundles in invasive foot structures termed invadopodia, which may confer increased metastatic potential in cancer cells [14–16]. Fascin is regulated by slug along with EMT and promotes intercalation of filopodia into mesothelial cell layers and cell invasion [17]. Recently, three groups found that NF-κB is required for the expression of Fascin in metastatic cancer cells [18–20], indicating that NF-κB may mediate a metastatic phenotype by specifically regulating Fascin.

The tumor suppressor p53 is widely known for its potential to induce cell death or cell cycle arrest and thereby prevent neoplastic progression [21, 22]. Deficiency or mutation of p53 commonly occurs in approximately half of all human cancers and contributes to tumor progression [23, 24]. Recently, p53 is shown to be associated with the process of EMT. p53 can regulate EMT and stemness or differentiation plasticity through activation of the p53-miR-200c pathway [25]. p53 is also demonstrated to control cancer cell invasion by inducing the MDM2-mediated degradation of Slug [26]. Consistent with the view that Slug can promote cancer cell invasion, Fascin is also shown to facilitate cell migration and invasion *in vitro* [15]. However, the role of p53 in regulating Fascin is not understood.

Here we report a new mechanism by which p53 regulates cancer invasion. It was demonstrated that wild-type p53 was able to inhibit cancer cell invasion by suppressing NF-κB-mediated activation of Fascin, whereas, p53 deletion triggered NF-κB-mediated activation of Fascin, thereby augmenting cancer cell invasion and metastasis. Furthermore, we showed that NF-κB was a key determinant for p53 deletion-mediated the up-regulation of Fascin.

## RESULTS

### Mutant p53 and Fascin expression correlate with poor survival time and distant metastasis of the patients with colorectal adenocarcinomas

**Figure 1:**
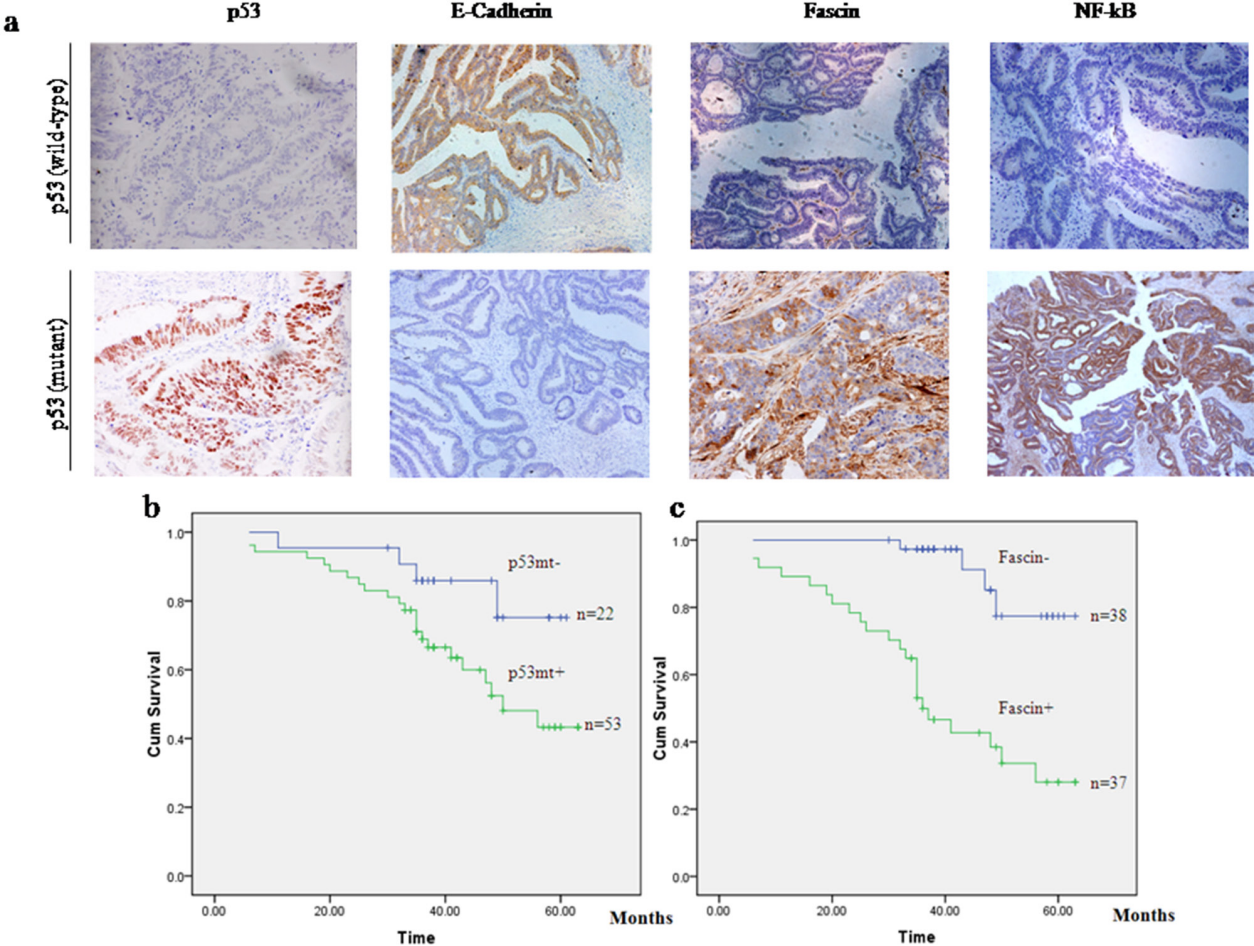
p53 mutation and Fascin expression are associated with poor clinical outcome in patients with colorectal adenocarcinomas **a.** Immunohistochemistry of E-cadherin, Fascin and NF-κB in serial sections of colorectal adenocarcinoma specimens with wildtype or mutant p53. The original magnification: × 200. **b.** Kaplan–Meier plots of overall survival for patients with colorectal adenocarcinomas with (p53mt^+^) or without (p53mt^−^) p53 mutation. **c.** Kaplan–Meier plots of overall survival for patients with colorectal adenocarcinomas with (Fascin^+^) or without (Fascin^−^) Fascin expression.

It has been acknowledged that mutant p53 can gain functions that accelerate malignant progression and increase cancer invasiveness and metastasis [27, 28]. To evaluate the role of mutant p53 and Fascin in colorectal cancer progression, we collected a cohort of 75 colorectal adenocarcinoma patients and determined the expression of p53 protein and Fascin by immunohistochemistry, as well as the p53 mutation status by direct DNA sequencing (Fig. 1a). The correlations between clinicopathological signatures of the patients with colorectal adenocarcinomas and p53 expression are shown in Table 1. Among these patients, 53 samples (70.7%) harbored mutant p53 protein expression which correlated with more distant metastasis (Table 1) and poor overall survival of patients with colorectal adenocarcinomas (Fig. 1b). Fascin was highly expressed in 49.3% (37/75) of colorectal cancer tissues and its expression was remarkably correlated with high tumor stage (Table 1) and poor overall survival of the patients with colorectal adenocarcinomas (Fig. 1c). Taken together, these results show that mutant p53 and Fascin are associated with increased risk of metastasis and mortality in colorectal adenocarcinomas.

**Table 1:** Correlation between clinicopathological background and p53 mutation status as well as NF-κB, Fascin, and E-cadherin expression in tumor specimens from 75 colorectal adenocarcinoma patients

Characteristics		Patient No.	p53 mutation		*P*-value^†^	NF-κB expression		*P*-value^†^	Fascin expression		*P*-value^†^	E-cadherin expression		*P*-value^†^
			Negative	Positive		Low	High		Low	High		Low	High	
Total No.		75	22	53		47	28		38	37		60	15	
Gender	Male	42	12	30	0.870	24	18	0.265	21	21	0.896	34	8	0.816
	Female	33	10	23		23	10		17	16		26	7	
Age	<60	37	8	29	0.148	22	15	0.571	17	20	0.420	31	6	0.419
	≥60	38	14	24		25	13		21	17		29	9	
pT categories	pT1–2	18	6	12	0.669	14	4	0.128	15	3	**0.001**	12	6	0.105
	pT3–4	57	16	41		33	24		23	34		48	9	
pN categories	pN0	32	12	20	0.180	21	11	0.648	22	10	**0.007**	22	10	**0.036**
	pN1/2	43	10	33		26	17		16	27		38	5	
pM categories	pM0	66	22	44	**0.039**	42	24	**0.003**	38	28	**0.001**	51	15	0.110
	pM1	9	0	9		1	8		0	9		9	0	
Stage-Dukes	B	29	12	17	0.053	20	9	**0.009**	23	6	**<0.001**	19	10	**0.030**
	C	37	10	27		22	15		15	22		32	5	
	D	9	0	9		1	8		0	9		9	0	

*High expression, more than 50%; low expression, 50% or less.

† Pearson Chi-Square test.

### Mutant p53 is associated with high expression of Fascin and low expression of E-cadherin in colorectal adenocarcinoma samples

To determine the relationship between mutant p53 and cancer-invasion-related genes, we further investigated the expression of Fascin and E-cadherin in colorectal adenocarcinoma specimens by immunohistochemistry (Fig. 1a). As a result, most tumors with mutant p53 were associated with low expression of E-cadherin (53/53, 100%) and high expression of Fascin (32/53, 60.4%). By contrast, the tumors with wild-type p53 were associated with high expression of E-cadherin and low expression of Fascin (Table 2), indicating that mutant p53 may induce a gain-of-function metastatic phenotype by regulating Fascin and E-cadherin.

**Table 2:** Relationship between p53 mutation status and the protein expression of p53, NF-κB, Fascin, and E-cadherin in tumor specimens from 75 colorectal adenocarcinoma patients

	Protein expression	p53 mutation		*P*-value^†^
		Positive (*n* = 53)	Negative (*n* = 22)	
p53	High	31 (58.5%)	6 (27.3%)	<0.05
	Low	22 (41.5%)	16 (72.7%)	
NF-κB	High	24 (45.3%)	4 (18.2%)	<0.05
	Low	29 (54.7%)	18 (81.8%)	
Fascin	High	32 (60.4%)	5 (22.7%)	<0.05
	Low	21 (39.6%)	17 (77.3%)	
E-cadherin	High	0 (0%)	15 (68.2%)	<0.05
	Low	53 (100%)	7 (31.8%)	

*High expression, more than 50%; low expression, 50% or less.

† Pearson Chi-Square test.

### p53 and Fascin correlate with colorectal cancer cell invasion and migration *in vitro*

**Figure 2:**
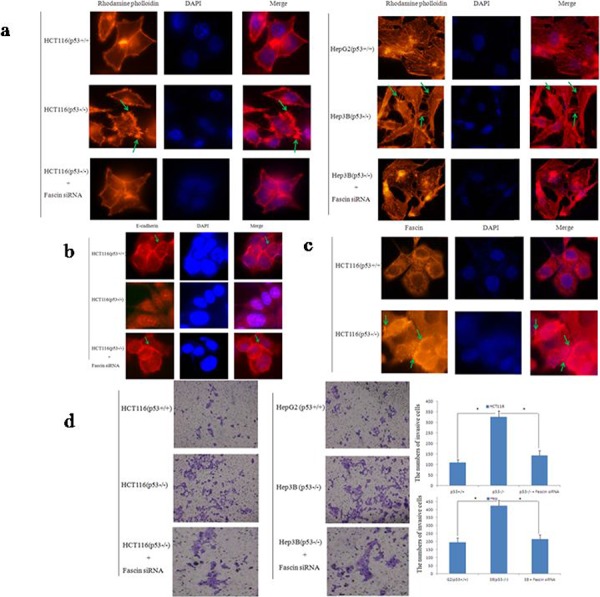
p53 and Fascin correlate with colorectal cancer cell migration *in vitro* **a.** Cell morphology and cytoskeleton analysis by immunofluorescence microscopy. Green stars indicate filopodia. The experiments were performed in triplicate. **b.** Representative images of E-cadherin expression in HCT116 cells. Photos were taken under × 400 magnification. Green stars indicate Fascin wxpression. The experiments were performed in triplicate. **c.** Representative images of Fascin expression in HCT116 cells. Photos were taken under × 400 magnification. Green stars indicate Fascin wxpression. The experiments were performed in triplicate. **d.** Transwell invasion assay by the 24-transwell system and quantitative analysis. The pictures were taken 24 h after seeding (original magnification: × 100). The numbers of invasive cells were counted in five representative high power fields per transwell. Three independent experiments were carried out in triplicate.

To evaluate the effect of p53 and Fascin on cell migration, we first examined the actin dynamics by rhodamine-conjugated phalloidin staining. It has been acknowledged that increased the filopodia formation enhances cancer cell invasion [29]. Intriguingly, we found that filopodia were significantly more frequent, longer in p53-deficient cells (HCT116 p53^−/−^ and HepG2) than wtp53 cells (HCT116 p53^+/+^ and Hep3B) (Fig. 2a), indicating p53 deletion promoted cancer cell invasion and migration. Next, immunofluorescence analysis was performed to examine the expression of E-cadherin and Fascin in HCT116 p53^−/−^ cells. The result revealed that wtp53 increased the expression of E-cadherin (Fig. 2b) and reduced the Fascin expression (Fig. 2c). In further confirmation of the negative effect of wtp53 on cell invasive migration, transwell assay was performed. As shown in Fig. 2d, p53 expression significantly suppressed colorectal cancer cell migration after 24 h incubation. Moreover, Fascin knockdown using siRNA in p53^−/−^ cells could rescue their morphology and migratory potential. These results indicate that wtp53 could suppress cell invasion and migration via inhibiting Fascin expression.

### Wild-type p53 inhibits Fascin protein expression

**Figure 3:**
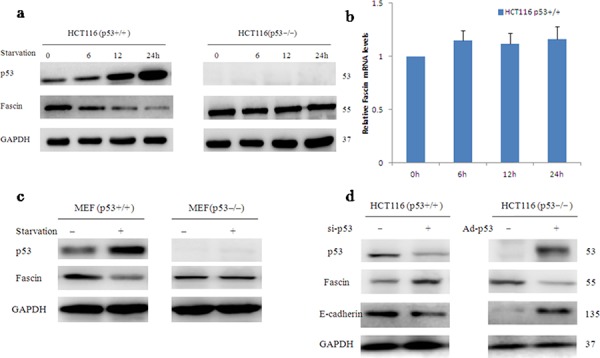
Wild-type p53 inhibits Fascin protein expression **a–c.** Glucose deprivation–induced p53 activation decreases the level of Fascin protein but not that of Fascin mRNA (the blots were cropped, and the full-length blots are included in the supplementary information). **d.** The effect of p53 on Fascin expression when the cells were transfected with p53-siRNA or p53 adenovirus plasmid.

Thus far, little is known about transcriptional regulation of Fascin. To examine whether Fascin expression is suppressed by p53 in response to stimuli, we subjected HCT116 p53^+/+^ to treatment with glucose deprivation. At 12 h after the deprivation of glucose, Fascin protein levels were significantly downregulated in parallel with upregulated wtp53 accumulation (Fig. 3a), but Fascin mRNA levels did not change (Fig. 3b), suggesting that Fascin is not transcriptionally regulated by wtp53. By contrast, the effects of glucose deprivation on Fascin levels were abolished in p53^−/−^ mouse embryo fibroblasts (MEFs) (Fig. 3c), indicating that Fascin downregulation is through wtp53. For further confirmation, we used short interfering RNA (RNAi) to knock down p53 expression in HCT116 p53^+/+^ cells and transfected HCT116 p53^−/−^ cells by p53 adenovirus plasmid. As shown in Fig. 3d, Knockdown of wtp53 increased Fascin expression, conversely, restoration of wtp53 caused a decrease in Fascin protein levels. These data suggest that wtp53 suppressed Fascin protein expression.

### p53 deletion stimulates Fascin expression via the NF-κB signaling pathway

**Figure 4:**
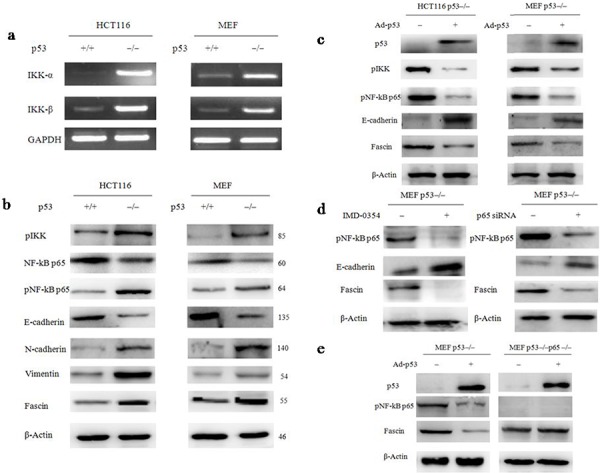
NF-κB is a key determinant for p53 deletion-mediated the up-regulation of Fascin **a.** The expression of IKKα and IKKβ, typical NF-κB target genes, in wild-type and p53^−/−^ HCT116s and MEFs was examined by RT-PCR. **b.** The expression of several key NF-κB signals regulators, E-Cadherin, N-Cadherin, Vimentin and Fascin were examined by western blotting (the blots were cropped, and the full-length blots are included in the supplementary information). **c.** The expression of pIKK, pNF-κB p65, E-Cadherin and Fascin in p53^−/−^ cells after the cells were transfected with p53 adenovirus plasmid. **d.** The expression of pNF-κB p65 and Fascin in p53^−/−^ MEFs after the cells were treated with the compound IMD-0354 or p65 siRNA. **e.** p53 deletion-induced Fascin activation is NF-κB-dependent. p53^−/−^ and p53^−/−^p65^−/−^ MEFs were infected with a p53 adenovirus plasmid for 48 h, and cell lysates were analysed by western blotting with the indicated antibodies (the blots were cropped, and the full-length blots are included in the supplementary information).

NF-κB has been demonstrated to contribute to metastatic phenotypes by specifically regulating Fascin [30, 31]. To investigate whether canonical NF-κB signal is a key determinant for p53 deletion-stimulated the Fascin expression, we first assessed the activation of two typical NF-κB target genes IκB kinase α (IKKα) and IκB kinase β (IKKβ) by RT-PCR and western blotting. We found that the activity of IKKα/β was remarkably increased in p53^−/−^ cells, compared with wtp53 cells (Fig. 4a), suggesting that the upstream of NF-κB signal was activated in p53^−/−^ cells. Next, the abundance of NF-κB was examined by western blotting (Fig. 4b). The result confirmed that NF-κB was significantly activated in p53^−/−^ cells, accompany with the down-regulation of epithelial marker E-cadherin and the up-regulation of mesenchymal markers N-cadherin, Vimentin and Fascin, indicating that p53 deletion activated NF-κB signaling pathway and triggered EMT transition which facilitated cancer cell invasion and migration. In the further confirmation, p53^−/−^ cells were transfected with a p53 adenovirus for 48 h. As a result, exogenous expression of wtp53 suppressed NF-κB activation and decreased Fascin level in p53^−/−^ cells (Fig. 4c).

To determine whether Fascin expression is regulated by p53 via NF-κB signaling pathway, we specifically attenuated NF-κB activation in p53^−/−^ MEFs using IMD-0354 (10 μM), a selective inhibitor of IKK-2 that is necessary for induction of NF-κB p65 nuclear translocation [32]. The inhibition of NF-κB by IMD-0354 suppressed Fascin expression in p53^−/−^ MEFs (Fig. 4d). We also performed p65 knockdown using siRNA in p53^−/−^ MEFs and obtained similar results (Fig. 4d). Moreover, exogenous expression of wtp53 decreased Fascin levels in p53^−/−^ MEFs but not in p53^−/−^p65^−/−^ MEFs (Fig. 4e), indicating that wtp53 may act inhibit Fascin activation through the suppression of NF-κB signal.

### The signature of p53 mutation, high NF-κB and Fascin, and low E-cadherin expression correlates with poor overall survival and early metastasis in patients with colorectal adenocarcinomas

**Figure 5:**
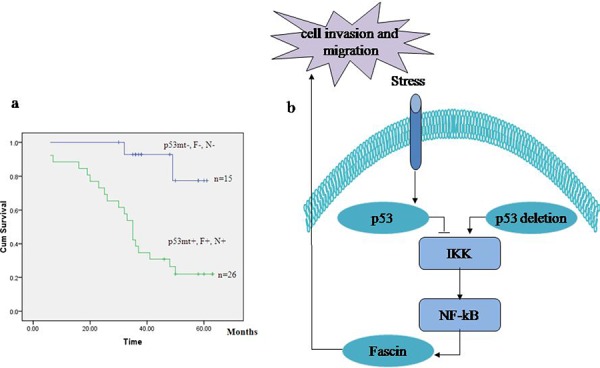
The signature of p53 mutation, high NF-κB and Fascin and low E-cadherin expression in patients with colorectal adenocarcinomas **a.** Kaplan–Meier analysis of overall survival for patients with colorectal adenocarcinomas with p53mt^+^ − NF-κB^+^ − Fascin^+^ versus p53mt^−^ − NF-κB^−^ − Fascin^−^. **b.** Model of the proposed link between p53, NF-κB, Fascin and cell invasion.

As shown in Table 2, the patients with p53 mutation showed high NF-κB and Fascin expression and low E-cadherin expression in colorectal adenocarcinomas, whereas wt p53 was correlated with low NF-κB and Fascin expression and high E-cadherin expression. To determine whether p53 mutation is related with the expression profiles of NF-κB and Fascin, we analyzed the prognostic significances of p53 mutation status, NF-κB and Fascin expression in 75 patients with colorectal adenocarcinomas by Kaplan–Meier analysis and log-rank test. As a result, the patients with a p53mt^+^ − NF-κB^+^ − Fascin^+^ expression profile had a significantly poorer overall survival than the p53mt^−^ − NF-κB^−^ − Fascin^−^ group (Fig. 5a). Thus, our results indicate that p53 mutation and high expression of NF-κB and Fascin hold potential as prognostic markers in colorectal adenocarcinomas.

## DISCUSSION

It is increasingly appreciated that tumor suppressor gene p53 plays a functional role in tumor initiation and progression, however, the function of p53 in tumor metastasis and EMT has not been well elaborated. In this study, we show a novel mechanism by which p53 inhibits colorectal cancer cell invasion through negative regulation of Fascin, a metastatic phenotype marker. We report that p53 mutation is correlated with low E-cadherin expression and high Fascin expression, as well as with poor overall survival. Moreover, p53 deletion promotes epithelial marker E-cadherin expression and decreases mesenchymal marker Fascin level, indicating p53 deletion triggers EMT transition and enhances colorectal cancer cell invasion and migration. In agreement with above results, the transwell assay also shows that Loss of p53 promotes the invasion and metastasis ability of colorectal cancer cells.

Emerging evidence has suggested that NF-κB pathway plays an important role in cell proliferation, motility, apoptosis, metabolism and DNA repair [33, 34]. Recently, several researches indicate NF-κB signals play an important role in the regulation of Fascin, however, whether Fascin expression is regulated by p53 via NF-κB signaling pathway remains unresolved. In the present study, we demonstrate that p53 deletion stimulates NF-κB activation and high Fascin level in cancer cells. Exogenous expression of wtp53 inhibits this effect. In further analysis, we show that NF-κB activation is a key determinant in p53 deletion-activated Fascin expression. The inhibition of NF-κB by the compound IMD-0354 or p65 siRNA suppresses Fascin expression in p53^−/−^ MEFs, moreover, exogenous expression of wtp53 decreases Fascin levels in p53^−/−^ MEFs but not in p53^−/−^p65^−/−^ MEFs. Taken together, our results demonstrate that p53 can suppress colorectal cancer cell invasion by inhibiting the NF-κB-induced activation of Fascin.

In conclusion, our results provide a new negative-feedback molecular mechanism by which loss of p53 facilitates cell invasion and migration via NF-κB-mediated activation of Fascin (Fig. 5b). This finding may offer new prognostic markers for the patients with colorectal adenocarcinomas.

## MATERIALS AND METHODS

### Cell lines, reagents and tumor samples

The human colorectal carcinoma HCT116 p53^+/+^, HCT116 p53^−/−^ cell lines were kindly given by Mian Wu. p53^+/+^ and p53^−/−^ MEFs were established from embryos of the corresponding mice generated by crossing p53 heterozygote mice (B6.129S2-Trp53tm1Tyj/J, The Jackson Laboratory, Bar Harbor, ME). HepG2 and Hep3B cells were purchased from ATCC (LGC Standards SLU, Barcelona, Spain). The cell lines were maintained in McCoy's 5A or Dulbecco's modified Eagle's medium (DMEM; Gibco BRL, Rockville, MD, USA) with 10% fetal bovine serum (FBS), 100 units/mL penicillin, 100 μg/mL streptomycin (Invitrogen), and 2 mmol/L L-glutamine at 37°C in a humidified atmosphere of 95% air and 5% CO_2_. The IKK-2 inhibitor IMD-0354 was purchased from Sigma Aldrich, St Louis, MO.

A total of 75 colorectal adenocarcinoma patients who underwent surgery between February 2004 and June 2006 at the Sir Run Run Shaw Hospital (Hangzhou, Zhejiang, China) were investigated. Patients who received pre-operative chemotherapy were excluded. This study was approved and monitored by the ethics committee of Sir Run Run Shaw Hospital, Zhejiang University.

### RNA extraction and RT–PCR

RNA was isolated from the cancer cells using a Qiagen RNAeasy kit (QIAGEN, Tokyo, Japan). Briefly, 1 μg of RNA was used to synthesize the first-strand cDNA using the Superscript system (Life Technologies, Inc.) in accordance with the manufacturer's instructions. RT–PCR reactions for IKKα and IKKβ were performed. For sequencing, the following primers were used: p53 Exon 5–8 forward 5′-TCTTCCTACAGTACTCCCCT-3′, reverse 5′-GCTTGCTTACCTCGCTTAGT-3′; p53 Exon 7–8 forward 5′-TAGGTTGGCTCTGACTGT-3′, reverse 5′-GCTTGCTTACCTCGCTTAGT-3′.

### RNA interference

Cells were transfected with p53 siRNA (Cell Signaling Technology, #6231) or NF-κB p65 siRNA (Cell Signaling Technology, #6261) via LipofectAMINE RNAi max (Invitrogen, 13778150) according to the manufacturer's instructions.

### Transwell invasion assay

Cells were trypsinized and resuspended in DMEM containing 1% fetal bovine serum at a density of 1 × 10^6^ cells/ml. 100 μl of the cell suspension was added into the upper chamber (was coated with Matrigel) of a transwell (Corning, Corning, NY, USA) consisted of inserts containing 8-μm pore-size PET membrane. DMEM (600 μl) containing 10% fetal bovine serum was placed in the lower chamber. After a 24 h incubation at 37°C cells remained in the upper chamber was removed carefully by cotton swab and the membrane was cut off by an operating knife. The side facing lower chamber was stained with 0.05% crystal violet and attached cells were counted under a light microscope. The experiment was performed three times.

### Immunofluorescence

Cells were cultured on glass coverslips and fixed in 3.7% paraformaldehyde in phosphate buffered saline (pH 7.4) for 10 min, permeabilized in 0.1% Triton X-100 in phosphate buffered saline for 4 min, blocked with 1% bovine serum albumin/phosphate buffered saline for 1 h, and then incubated at room temperature for 1 h with rhodamine-conjugated phalloidin (Invitrogen, Carlsbad, CA, USA) or Fascin at 1:100 in blocking solution. Nuclei were counterstained with DAPI. Images were acquired with an Olympus BX51 microscope.

### Antibodies

p53 antibody (DO-1): sc-126 were obtained from Santa Cruz Biotechnology (Santa Cruz, CA, USA). NF-κB p65 antibody (#8242), phospho-NF-κB p65 (Ser468) (#3039), Phospho-IKKα (Ser176)/IKKβ (Ser177) (C84E11) Rabbit mAb (#2078), E-Cadherin (24E10) (#3195), N-Cadherin (D4R1H) (#13116) and phospho-NF-κB p65 (Ser 536) (#3031) antibodies were obtained from Cell Signaling (Beverly, MA, USA). Fascin antibody (ab78599) was obtained from Abcam. The anti-β-actin antibody (AC-15) was purchased from Sigma-Aldrich (St. Louis, MO, USA).

### Immunohistochemistry staining

The ChemMate EnVision Detection Kit (DAKO, Carpinteria, CA, USA) was used for immunohistochemistry according to company's recommended procedure. Briefly, after being deparaffinized and hydrated, the paraffin-embedded sections were placed in 0.01M sodium citratebuffer (pH 6.0), and subjected to pressure cooker treatment for 2 min at full pressure with a domestic pressure cooker. After cooling to room temperature, the slides were rinsed with Tris buffered saline (0.05 M Tris/0.15M NaCl, pH 7.6). The endogenous peroxidase activity was blocked by incubating the sections with 3% hydrogen peroxide. The sections were incubated with the primary antibody overnight at 4°C. Then, the ChemMate EnVision/HRP, Rabbit/Mouse (ENV) reagent was applied to the sections, followed by application of ChemMate DABt Chromogen included in the kit. The slides were lightly counterstained with hematoxylin.

### Western blot analysis

Cells were harvested from cultured dishes and were lysed in a lysis buffer [20 mM Tris-HCl pH 7.6, 1 mM EDTA, 140 mM NaCl, 1% NP-40, 1% aprotinin, 1 mM phenylemethylsulfonyl fluoride (PMSF), 1 mM sodium vanadate]. Protein concentration was determined using a BCA Protein Assay Kit (Pierce). Cell lysates (40 μg protein/line) were separated on a 5 to 20% Tris-Tricine Ready Gel SDS-PAGE (Bio-Rad) for nitrocellulose membrane blotting. The blotted membranes were blocked with 5% skim milk for 1 h and were incubated with primary antibodies. The immunoreactive bands were visualized by enhanced chemiluminescence using horseradish perox-idase-conjugated IgG secondary antibodies. Band density was measured by densitometry, quantified using gel plotting macros of NIH image 1.62, and normalized to an indicated sample in the identical membrane.

### Statistical analyses

Results are expressed as values of mean ± standard deviation (SD). Statistical analysis was performed using SPSS 16.0 for Windows (SPSS Inc., Chicago, IL, USA). The correlation coefficient of two factors was evaluated using Chi-square and Fisher's exact tests. The survival of patients with colorectal adenocarcinomas was compared using the Kaplan–Meier method, and differences between the survival curves were tested using the logrank test. A *P*-value less than 0.05 is considered significant.
